# Municipal chief nurses’ responsibilities and decision-making to ensure patient safety in municipal healthcare: a qualitative descriptive study

**DOI:** 10.1186/s12912-025-03295-3

**Published:** 2025-06-04

**Authors:** Elisa Reinikainen, Annica Kihlgren, Margaretha Norell Pejner, Jenny Windahl

**Affiliations:** 1 Örebro University, Fakultetsgatan 1, Örebro, SE-701 82 Sweden; 2 Halmstad Municipality, Halmstad, Sweden

**Keywords:** Decision-making, Municipal healthcare, Municipal chief nurse, Patient safety

## Abstract

**Background:**

The structure and organisation of Swedish municipal healthcare has been criticised for being fragmented and inefficient. Municipal chief nurses hold overall responsibility for patient safety within municipal healthcare, yet they lack authority over finances and staffing. Their role and responsibilities have not been examined in previous studies. The aim of this study was therefore to explore municipal chief nurses’ experiences of obstacles and possibilities in decision-making to ensure patient safety in municipal healthcare.

**Methods:**

A qualitative descriptive design was used. Data were collected through 15 individual semi-structured interviews with municipal chief nurses, and the results were analysed via qualitative content analysis.

**Results:**

The data analysis yielded an overall theme: Navigating decision-making to ensure patient safety. This overall theme comprised three underlying categories: Unclear role and understanding of the assignment; Impact of organisational level on decision-making mandate; and Knowledge, competence, and experience in patient safety work.

**Conclusion:**

There was a lack of clarity regarding the municipal chief nurses’ assignments in the municipal healthcare organisation. The participants felt that their organisations had insufficient knowledge of healthcare, and it became evident that organisational placement and their own competence affected their decision-making regarding healthcare and patient safety. Some noted that the organisation’s shortcomings could be advantageous, providing them with a scope for action. Consequently, the informants had to navigate in the system and find alternative ways to ensure patient safety.

**Clinical trial registration:**

Not applicable.

## Background

Patient safety is a global concern [1, 2]. Annually, there are 134 million recorded adverse events in healthcare, resulting in 2.6 million deaths [2]. In a broader context, patient safety encompasses a framework of organised activities aimed at lowering risks, minimising errors, and mitigating harm when it does occur within healthcare environments. Strategy 5.3 of the World Health Organization (WHO) Global Patient Safety Action Plan 2021–2030 emphasises the importance of incorporating patient safety core competencies into the regulatory requirements for health professionals [2]. Depending on the target group of patients, care facilities and services can be hospitals, health centres, homes, or special accommodations.

In Sweden, the responsibility for healthcare for older persons is shared between the regions and the municipalities [3]. Municipalities are tasked with providing social services and healthcare up to and including the level of Registered Nurses, while the regions are responsible for delivering medical care. This necessitates close collaboration between healthcare providers. To ensure patient safety and quality work within the municipality, legislation mandates that each municipality must have a municipal chief nurse (MCN) [3].

The municipalities have autonomy in decision-making regarding routines and policy issues in municipal healthcare. The responsibilities of MCNs are legally regulated, and encompass establishing routines and guidelines for medication management, delegation, documentation, deviation management, and procedures for contacting physicians [4]. While the MCNs hold overall responsibility for patient safety in nursing, they lack budgetary or personnel authority to implement their decisions. This responsibility lies at various organisational levels within the municipality, including the political board, the head director of administration, the operational manager, and the first-line managers for all clinical care units.

The current organisational structure, where the MCN is accountable for patient safety without financial or personnel authority, has faced criticism [5]. This type of organisation requires close cooperation between healthcare providers [6]. According to the WHO, all levels within the organisation must collaborate to enhance patient safety [7]. A lack of close collaboration, especially regarding financial or personnel resources, can jeopardise patient safety [8].

Managers’ attitudes significantly impact patient safety [9, 10]. For example, when management promotes the reporting of patient safety deviations to improve healthcare, patient safety is enhanced [11]. Managers’ willingness to alter the safety climate is evident in their interactions and communication with staff [12]. These responsibilities demand a high level of competence, defined as the ability to satisfactorily perform tasks in specific situations [13].

Leadership or organisations that lack resources, support, and knowledge, or fail to acknowledge the connection between leadership and patient safety, risk compromising patient safety [8]. Tensions between decision-makers and managers can jeopardise patient safety, highlighting the need for structured routines and frameworks [14]. Training and skills development are crucial factors that positively impact patient safety [8]. Leadership and organisational efforts can significantly contribute to the creation of a patient-safe approach by promoting employee ownership and encouraging innovative work [8]. Thorough planning and implementation of strategies to enhance care quality and ensure a safe environment for patients are vital elements of a patient safety leadership initiative [15].

In summary, the MCN’s additional responsibilities include inspection, follow-up, reporting of patient safety shortcomings, and directives for improvements in health and medical care [3, 4]. Although the MCN is responsible for patient safety, they lack the staff and financial resources to implement their decisions regarding guidelines or procedures to ensure patient safety. The implementation of patient safety measures falls to the political board, the head of administration, the operational manager, or first-line-managers. Meanwhile, the MCN collaborates closely with other healthcare providers, such as the regions, that are also involved in patient safety efforts. To ensure patient safety, the MCN must find alternative approaches. The aim of this study was therefore to explore MCNs’ experiences of obstacles and possibilities in decision-making to ensure patient safety in municipal healthcare.

## Method

### Design

A qualitative descriptive design was used to gain a deeper understanding of the experiences of MCNs in ensuring patient safety without having mandated decision-making. Data were gathered via semi-structured interviews and analysed via qualitative content analysis [16].

### Setting

The study was conducted in 2021 in a large region of Sweden that is divided into several municipalities. MCNs from 12 municipalities were included in the study. During the year of the study, a total of 13 526 persons in these 12 municipalities received municipal health and medical care (per-municipality range: 127–7515, mean: 1127, median: 462) [17].

### Participants

Participants were strategically selected to ensure a range of experience levels as MCNs. Inclusion criteria required participants to have worked as an MCN for approximately two years or more, with experience predating the COVID-19 pandemic.

Based on the inclusion criteria, 15 participants were recruited through a network for MCNs across various municipalities in the region. They were contacted by e-mail, provided with written information about the study, and invited to participate. The information specified that participation was voluntary and that they could withdraw from the study at any time. Before deciding whether to participate, they were given an opportunity to ask questions. Two MCNs did not respond to the invitation, so two additional MCNs were contacted, both of whom agreed to participate.

### Data collection

The first author gathered data through individual semi-structured interviews [16]. Participants were contacted via e-mail to schedule the interviews. Due to national restrictions in Sweden during the COVID-19 pandemic, the interviews were conducted digitally using Teams, a digital communication application.

The authors developed a semi-structured interview guide for this study (see Additional file 1). The interview guide included open questions about experiences working as an MCN, possibilities and obstacles in fulfilling responsibilities, decision-making mandates, and organisational placement. Probing questions such as ‘What do you mean by that…’, ‘Can you elaborate…’, ‘What do you think it depends on… ’, and ‘How does that affect your work…’ were used to obtain a deeper understanding of their experiences. Open questions were used to allow the participants to talk freely about their experiences using their own words [18].

The interview guide was pilot tested in interviews with three of the MCNs who consented to participate. The questions were assessed to be relevant and comprehensible; therefore, no alterations were made to the interview guide, and the pilot interviews were included in the main study. Data saturation was achieved after 15 interviews, as by this point little or no new information was being obtained [19]. The interviews were recorded using a tape recorder and transcribed verbatim by the first author.

### Data analysis

The transcribed data were analysed using qualitative content analysis [16]. Each author individually read through the transcribed interviews multiple times to gain a deeper understanding of the content, which was then collectively discussed on several occasions. Sentences or paragraphs with similar meanings were extracted and condensed into meaning units. Next, the codes were compared based on differences and similarities and sorted into six subcategories. These subcategories were then grouped into three categories which were combined to interpret the results. One theme emerged, illuminating the underlying meaning of the meaning units, codes, subcategories, and categories. Although the process is described linearly, the analysis involved a back-and-forth movement between different parts of the text. Examples from the data analysis process are presented in Table 1.

**Table 1:** Examples of the analysis process with coding, subcategorising, and categorising of the interview data

Interview text	Meaning unit	Code	Subcategory	Category	Theme
*“The role of MCNs is not particularly regulated*,* and what is outlined in the healthcare regulations represents only a small portion of our actual responsibilities. Our role varies significantly across different municipalities*,* and the nature of our assignments differs because there is no standardised regulation.”* (MCN 12)	The assignments of MCNs vary, causing confusion and affecting their decision-making authority. The role of MCNs is not fully regulated.	An ambiguous assignment	Different interpretations of the assignment	Unclear role and understanding of the assignment	Navigating decision-making to ensure patient safety
*“The further down you are in the organisation*,* the more difficult it becomes to make decisions and to have the courage to implement changes. Conversely*,* if you are higher up*,* such as just below the Head of Social Services*,* it is easier to accomplish certain tasks to ensure patient safety.”* (MCN 2)	The possibilities and decision-making mandates of MCNs are influenced by their organisational placement and their collaboration with management.	Organisational placement	Organisational placement affects patient safety	Impact of organisational level on decision-making mandate	
*“There is a lack of genuine understanding of healthcare. I have to fight hard for patient safety and healthcare issues.”* (MCN 4)	A limited understanding of healthcare issues makes it challenging for MCNs to explain patient safety within the organisation	Knowledge of healthcare is lacking within the organisation	Competence in the organisation	Knowledge, competence, and experience in patient safety work.	

MCN: municipal chief nurse

### Ethical considerations

The study was approved by the Swedish Ethical Review Authority (registration numbers 2021 − 01862, 2022-04546-02, 2022-07330-02) and was performed in accordance with the Swedish Act concerning the Ethical Review of Research Involving Humans. The study also followed the Declaration of Helsinki. Participants received written and verbal information about the study, and they were also informed that participation was voluntary and that they could withdraw at any time. Written informed consent was obtained from the study participants prior to the interviews.

The participants’ identities were known to the interviewer. To ensure confidentiality during data handling and analysis, each respondent’s transcription was assigned a code number. This ensured that the reporting of study results and the quotations presented did not compromise the participants’ anonymity. Recorded interviews were stored in a locked safe, separate from the code list.

## Results

The collected data consisted of 317 min of recorded interviews and 66 pages of transcribed text. Each interview lasted between 15 and 39 min. The study participants had been working as MCNs for between two and 18 years (median: six years). Nine of the participants had a specialist nurse examination in primary healthcare or similar, and 10 had received some form of leadership education. Within the organisational structure, nine of the participants were placed under the head director of administration, four were placed under the quality manager, and two were placed under a first-line manager.

**Fig. 1:**
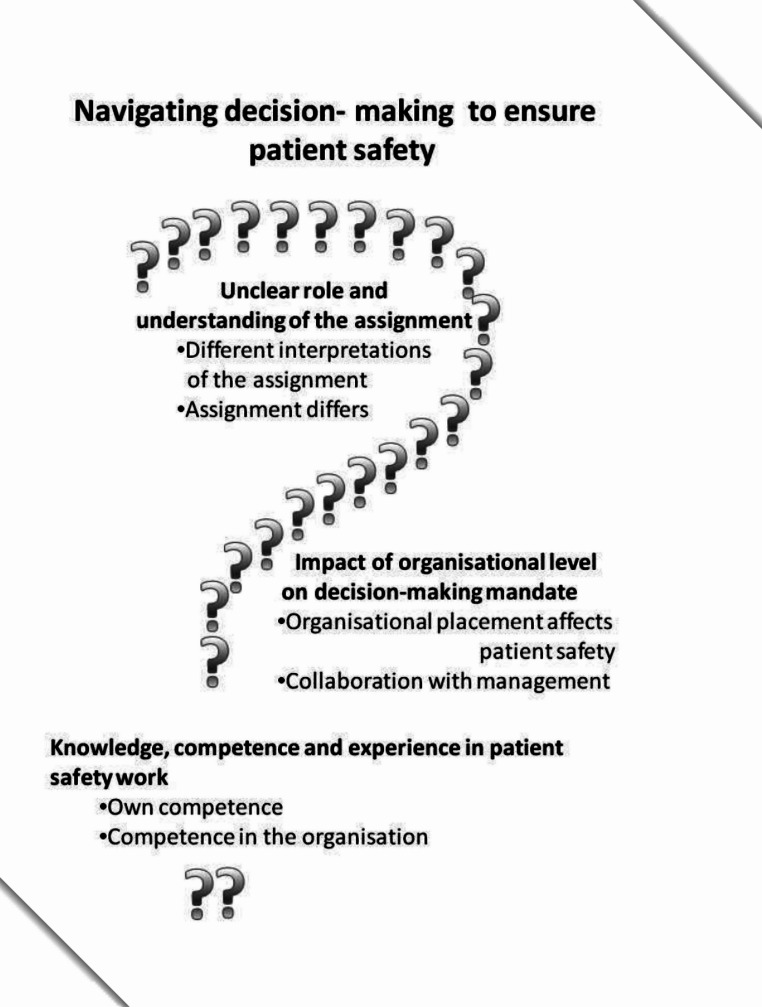
Overview of the theme with categories and corresponding subcategories

The data analysis resulted in an overall theme: *Navigating decision-making to ensure patient safety.* This overall theme comprised three underlying categories: *Unclear role and understanding of the assignment*; *Impact of organisational level on decision-making mandate*; and *Knowledge*,* competence*,* and experience in patient safety work* (Fig. 1).

The overall theme outlines the challenges the MCNs encountered in decision-making to ensure patient safety. It highlights the lack of clarity regarding their assignments and the implementation of patient safety decisions within the organisation. Despite these ambiguities, both barriers and opportunities in patient safety work were described.

### Unclear role and understanding of the assignment

This category explores issues arising from the unclear mission of the MCNs’ role and its impact on their decision-making mandate. Two subcategories highlight the factors contributing to the ambiguity of the MCNs’ role: *Different interpretations of the assignment* and *Assignments differ*.

#### Different interpretations of the assignment

Municipal management determined the responsibilities of the MCNs, resulting in different interpretations of the assignment. This appeared to vary across municipalities. One MCN said:

The MCN’s role is not particularly regulated, and what is stated in the Health and Medical Services Act is just a tiny part of what we actually do, in my experience. There is no clear guidance, and the MCN role varies greatly between different municipalities, depending on the specific tasks assigned. (MCN 12)

#### Assignments differ

In addition to their statutory assignments, the MCNs were also expected to undertake additional tasks such as educating healthcare staff and advising social workers on individual cases. They were also sometimes responsible for drafting responses to referrals, managing service procurement requirements, and authorising access to digital systems. Some MCNs noted that management was unsure how to effectively utilise their role. One stated:

I try to fight against being involved with everything. I must take care of the things I am responsible for. I’d like to take care of everything, but it’s just not possible. (MCN 9)

Several MCNs expressed that their roles were ambiguous, which they found problematic. They experienced uncertainty regarding their responsibilities and the scope of their duties and mandates. This lack of clarity hindered their decision-making. One MCN remarked:

The biggest obstacle is that people don’t understand the MCN role. (MCN 3)

Other MCNs viewed the lack of clarity in their role descriptions as an opportunity to shape their own assignments independently. They appreciated the freedom to plan their work and prioritise their responsibilities. One way to do this was by establishing a network within the organisation. However, newcomers to the role found it particularly challenging to understand their tasks and decision-making authority. Despite this, all MCNs expressed a desire for a more regulated mandate and clear role descriptions to ensure consistent across municipalities. One MCN suggested:

Strengthen the role of the MCNs to ensure patient safety. Legislate it differently so that each of the municipalities do it in the same way, that the roles of the MCNs are equal. (MCN 9)

### Impact of organisational level on decision-making mandate

This category explores how placement at higher organisational levels enhanced the MCNs’ decision-making authority and ability to fulfil their responsibilities. Two subcategories illustrate how these impacts could occur: *Organisational placement affects patient safety* and *Collaboration with management*.

#### Organisational placement affects patient safety

Most MCNs felt that being placed at a higher organisational level improved their ability to fulfil their responsibilities and strengthened their decision-making mandate. One MCN reflected:

Very good possibilities due to the organisational placement and the fact that I have the space to make decisions. I report directly to the social director and do not have a first-line manager as I did previously, which made it impossible for me to carry out my duties. (MCN 8)

Being placed at a higher organisational level gave the MCNs the opportunity to join the management team and engage in strategic decision-making related to healthcare and patient safety issues. For instance, they attended management team meetings and collaborated within the municipal association in the region. However, many MCNs did not participate in these meetings. Some mentioned that they were not invited to join strategic discussions between the municipalities on healthcare development and patient safety, or when various authorities wanted to address shortcomings in municipal homecare. One MCN stated that not being included in discussions about healthcare issues posed an obstacle to decision-making:

Various networks are created, but there are many social managers and municipal directors in the groups. However, there is no relation to the MCN as there was before; instead, healthcare is discussed by people who don’t have the formal competence to do so. (MCN 11)

#### Collaboration with management

Working in proximity to the management team facilitated the MCNs in their development of healthcare. For example, it eased communication when healthcare and patient safety work needed to be discussed, and it was also useful when they needed to explain the consequences of any shortcomings to the management team. The MCNs also experienced other problems in relation to their placement in the organisations. For example, some MCNs occasionally experienced a sense of awkwardness when their organisational position fell under the supervision of a first-line manager who was responsible for monitoring the same unit; they themselves were monitoring. This situation could create the perception that they were inspecting their own managers’ work. One MCN stated:

I’ve told my boss that I also review what you do. It’s like having one foot in two different camps. (MCN 4)

The MCNs found it challenging to address any identified issues with their first-line managers. These managers might not fully understand the impact of service shortcomings, and they lacked the authority to address patient safety issues as effectively as higher-level managers within the organisation. One MCN who had a higher-level organisational placement, under the Head of Administration, described their decision-making mandate as good, which strengthened their ability to carry out their responsibilities. One MCN stated:

The further down you are in the organisation, the more difficult it becomes to make decisions and to have the courage to implement changes. Conversely, if you are higher up, such as just below the Head of Social Services, it is easier to accomplish certain tasks to ensure patient safety. *(MCN 2)*

### Knowledge, competence, and experience in patient safety work

This category explores the significance of the MCNs’ expertise in leadership, municipal healthcare, laws, regulations, and organisational knowledge. It also highlights the impact of management’s limited understanding of the MCNs’ responsibilities and mandate. Two subcategories illustrate the contributions of the various actors within the organisation: *The MCN’s own competence* and *Competence in the organisation*.

#### The MCN’s own competence

This subcategory refers to the MCNs’ competence and experience in leadership, municipal healthcare, laws and regulations, social services, and organisational aspects of healthcare and municipalities which were essential for fulfilling their responsibilities. Their competence and prior experience instilled confidence in others, enhancing their reputation as trustworthy and reliable. This credibility earned them a mandate from management and first-line managers, providing better opportunities to fulfil their responsibilities. It enabled the MCNs to receive support in their work and invitations to management team meetings, allowing them to participate in and influence decisions related to patient safety in municipal healthcare. One MCN stated:

Being self-assured. That I can show that this is how it is, I am straight and consistent. Aware. That I have good knowledge and can give answers. It is a question of trust. (MCN 15)

#### Competence in the organisation

This subcategory conversely refers to management’s lack of knowledge and understanding of the MCNs’ responsibilities. This was seen as an obstacle, negatively impacting the mandate the MCNs received within the organisation. The MCNs stated that the absence of national competence requirements for their role hindered their ability to fully perform their responsibilities. Many MCNs also felt isolated in their roles due to the lack of colleagues with healthcare expertise to discuss important matters with. They perceived their assignment as relying heavily on their own experience, skills, and competence, often without organisational support. However, participating in the regional network offered valuable support from more experienced MCNs and facilitated discussions on national directives and guidelines. One MCN stated:

You sometimes feel vulnerability and loneliness when there are difficult decisions. It is you who must decide on certain issues; it can be very difficult. (MCN 7)

In many municipalities, first-line managers often had little or no healthcare education or experience. The MCNs perceived this as a significant obstacle, as the managers lacked the understanding necessary to take actions to improve patient safety. When no one in a leading position had healthcare education, they were unable to lead development efforts effectively, resulting in MCNs being assigned more responsibilities and mandates than their statutory responsibilities. The MCNs believed that if operational managers had healthcare education, they would be better equipped to contribute to the development of healthcare and patient safety in their units. One MCN stated:

I feel incredibly alone in having healthcare skills; I wish there was a manager who had healthcare skills at the leadership level. I feel my hands are tied; there is no one to discuss the benefits of certain interventions with. (MCN 5)

Some MCNs viewed the lack of healthcare education among managers as an opportunity, experiencing greater autonomy in their roles. This situation meant their work was not scrutinised, allowing them to operate more freely and independently.

## Discussion

The aim of this study was to explore MCNs’ experiences of obstacles and possibilities for decision-making to ensure patient safety in municipal healthcare. The results show a lack of clarity around MCNs’ role and decision-making on patient safety issues, requiring MCNs to navigate within the system and find alternative ways to ensure patient safety. This entailed both obstacles and opportunities for the MCNs in their patient safety work. The MCNs’ own understanding of their assignment, as well as the surrounding organisation’s understanding of the assignment, could be seen as an opportunity or an obstacle for their perceived mandate and decision-making abilities. The ambiguity of the MCNs’ assignment within the organisation could be perceived as an opportunity for them to independently perform their work. However, it became an obstacle when tasks beyond their statutory duties were added.

### Unclear role and understanding of the assignment

The MCN function was established after a political decision in Sweden to safeguard patient safety. While MCNs can intervene in individual patient cases, their overall responsibility is broader. The results show that the MCN’s mission varies across different municipalities, leading to a lack of clarity about what should be included in their role. Many MCNs find themselves having to take on additional tasks beyond their statutory responsibilities. According to Lipsky, individuals whose work is based on political decisions often face conflicts due to ambiguous decisions [20]. It is paradoxical that, despite the statutory nature of the MCNs’ tasks, their role is perceived as unclear. Nonetheless, the results show that organisations interpret their mission in various ways.

Some MCNs stated that the unclear mission gave them opportunities to be more independent. However, this necessitated the establishment of a network of relationships within the organisation. Cabral et al. have previously described how nursing leaders, who have a complex and demanding role in an organisation, need support from other people to be able to deliver results [21]. Having such support could also reduce the MCNs’ experience of isolation and insecurity in fulfilling their duties. According to Nilsson and Sandoff, building personal relationships is essential in organisations with deficiencies in the formal structure [22]. Moreover, a Swedish study found that MCNs’ personal relationships with nurses contributed to a higher willingness to report deviations, thereby enhancing patient safety work [11].

### Impact of organisational level on decision-making mandate

The results show that positioning MCNs at a higher organisational level facilitates the implementation of patient safety work. The organisational level is crucial for patient safety work, as it encompasses the entire organisation’s responsibility [23]. This responsibility includes understanding patient safety culture, policies, routines, and the impact of various decisions on patient safety. To enhance their role, MCNs need acknowledgment at both professional and organisational levels [24]. Additionally, placing MCNs at lower organisational levels often results in strategic healthcare decisions being made without medical expertise.

Beyond their placement in the organisational hierarchy, the organisational culture can negatively impact the role of MCNs and nurses as professionals [24]. Rihari-Thomas et al. have emphasised the need for a clear mandate within the organisation to effectively implement patient safety work [25]. The MCNs in the present study noted that they often lacked access to the organisation to participate in strategic decisions. Additionally, first-line managers may feel uncertain about their mandate concerning patient safety issues, further compromising patient safety [26]. This uncertainty affects collaboration both within their own organisation and in relation to the regions.

Lipsky suggests that granting mandates beyond legal requirements can lead to conflicts due to differing focuses [20]. For MCNs, this conflict may arise between maintaining a client-centred approach and aligning with the organisation’s overall goals. This can impact patient safety, as some issues are administrative, such as procuring technical equipment or establishing agreements with other healthcare providers regarding liability [27]. Although MCNs can highlight patient safety needs, they lack the authority and mandate to implement changes, despite their legal responsibility for patient safety.

### Knowledge, competence, and experience in patient safety work

Implementation of the MCNs’ decisions relied heavily on their own knowledge and experience. These attributes promoted employees’ trust in MCNs and provided the necessary legitimacy for patient safety work. However, the MCNs in this study did not specify the type of knowledge they were referring to. Heinen et al. have highlighted the importance of understanding how an organisation operates within its context, including knowledge of both one’s own organisation and the decision-making processes of partners [1]. Leadership knowledge is also crucial. A study in Norway found a connection between transformational leadership and an enhanced patient safety culture in nursing homes and home care settings [28]. Additionally, a study conducted in 12 European countries revealed that patient safety work was given higher priority when registered nurses held leadership positions [29].

Effective leadership is essential for ensuring the quality of care [30]. Emphasising leadership could be a way to increase patient safety. Regardless of their health and social care education, managers should possess the competence to support individuals in their creativity to achieve health and care goals in nursing homes [31]. The WHO emphasises that all healthcare professionals, managers, and leaders must understand patient safety [2]. This includes comprehending the nature and significance of risks, how harm occurs, key concepts in patient safety science, methods for investigating and understanding the causes of unsafe care, and the necessary actions to maximise patient safety.

### Methodological considerations

The qualitative approach of Graneheim and Lundman [16] was considered appropriate for investigating MCNs’ experiences and gaining a deeper understanding of the MCNs’ function within municipalities. Although one interview was brief, the richness and detail in the content of the analysed material were well suited to the aim of the study. A strength of the study lies in the rich and nuanced data acquired from the interviews. Having open-ended questions and the opportunity for the participants to orally describe the studied phenomena gave the authors valuable nuanced information from different perspectives. One author is currently employed as an MCN, and another author has previously worked as an MCN. This may be seen as a limitation of the study; however, it also allowed for a greater understanding of the concepts being explored. The analysis of the results may have been enriched by the fact that two of the authors have not worked as MCNs. To maintain confidentiality, the gender of the participants is not disclosed, which might be seen as a limitation. However, since most MCNs in Sweden are female, we chose to protect the anonymity of male participants.

Due to the COVID-19 pandemic, data collection was conducted via digital interviews. It is difficult to assess the extent to which the digitally conducted interviews may have negatively impacted the interaction with participants, compared to face-to-face interviews. However, the information the MCNs provided was rich, and they seemed to feel free to discuss their work. A limitation of the study might be that only one region in Sweden was included. Nevertheless, the results should be transferable to municipal healthcare both within Sweden and internationally, based on the rich information obtained from the interviews.

## Conclusions

The current study revealed a lack of clarity regarding MCNs’ assignments in the municipal healthcare organisation. The MCNs also felt that their organisations had insufficient knowledge of healthcare, and it became evident that organisational placement and their own competence affected their decision-making. Some MCNs noted that the organisation’s shortcomings in healthcare and patient safety could be advantageous, as it provided them with more room for action. Consequently, the MCNs had to navigate in the system and find alternative ways to ensure patient safety.

## Data Availability

The data (in Swedish) used and/or analysed during the current study are available from the corresponding author upon reasonable request.

## Abbreviations

MCN: Municipality chief nurse
WHO: World Health Organization
